# Relationship Between Subclinical Hypothyroidism in Pregnancy and Hypertensive Disorder of Pregnancy: A Systematic Review and Meta-Analysis

**DOI:** 10.3389/fendo.2022.823710

**Published:** 2022-03-08

**Authors:** Yue Han, Jun Wang, Xiaoying Wang, Ling Ouyang, Yan Li

**Affiliations:** Department of Obstetrics and Gynecology, Shengjing Hospital of China Medical University, Shenyang, China

**Keywords:** subclinical hypothyroidism, hypertensive disorders of pregnancy (HDP), levothyroxine alone, thyroid-stimulating hormone (TSH), pregnancy

## Abstract

**Objective:**

Studies have shown a high incidence of subclinical hypothyroidism in pregnancy, but the adverse pregnancy outcomes caused by it are not clear. Therefore, we conducted a systematic review and meta-analysis to evaluate the relationship between subclinical hypothyroidism in pregnancy and hypertensive disorders of pregnancy(HDP) to guide clinical practice.

**Method:**

We searched the MEDLINE (PubMed), Cochrane Central, EMBASE, Web of Science, and SCOPUS databases and screened all studies evaluating the relationship between subclinical hypothyroidism in pregnancy and hypertensive disorders of pregnancy. Two researchers independently evaluated the quality of all eligible original studies using the Newcastle-Ottawa Scale (NOS). We also performed a meta-analysis using STATA15.1. Sensitivity analyses were also performed by examining the effects of individual studies as well as using different effect models and detecting any publication bias using the harbord test.

**Results:**

Twenty-two studies were included in the final meta-analysis. Our results indicated that pregnant women with subclinical hypothyroidism had an increased risk of HDP (OR = 1.54(95% CI: 1.21-1.96) I²=67.1%), compared with euthyroidism. Subclinical hypothyroidism in pregnancy was not associated with hypertensive disorders of pregnancy at TSH diagnostic cut-off of less than 3.0 mIU/L (P = 0.077). Curiously, the risk of HDP increases when the TSH diagnostic cut-off value is higher or lower than 4 mIU/L. Although only 9 studies were above the threshold, the risk of developing HDP was still 1.69 times, which was highest in all subgroup analyses. This is consistent with the newly recommended diagnostic cut-off value of 4 mIU/L for TSH by the ATA. Our results consider that the risk of hypertensive disorder complicating pregnancy is increased regardless of the diagnosis of subclinical hypothyroidism at any stage of pregnancy. Unfortunately, there is insufficient evidence to support that patients can benefit from treatment with levothyroxine.

**Conclusion:**

The results of this meta-analysis indicate that subclinical hypothyroidism in pregnancy is associated with an increased risk of developing HDP, and this association exists regardless of the gestational period. However, the available evidence cannot support these patients receiving thyroxine intervention can benefit from it, so routine screening is only recommended for pregnant women with risk factors for hypothyroidism. Further research is needed to validate more scientific and rigorous clinical studies to clarify the relationship between subclinical hypothyroidism and HDP to improve patient prognosis.

**Systematic Review Registration:**

https://www.crd.york.ac.uk/prospero/, PROSPERO (CRD42021286405)

## Introduction

As one of the most important endocrine diseases in pregnant women, thyroid disease during pregnancy has gradually become a hot spot in clinical and basic research in the field of maternal-fetal medicine with the publication of the results of more than ten large-sample clinical trials in recent years. Among them, subclinical hypothyroidism as a population with a large number of patients has also attracted countless attention. Subclinical hypothyroidism (SCH) refers to elevated serum TSH levels with normal fT4 or TT4 values (1). According to incomplete statistics, 10% of adults, as well as 3.47% of pregnant women are currently afflicted (2, 3). However, individual differences and the presence of other confounding factors (such as iodine intake, thyroid antibody status, etc.) make the establishment of an appropriate reference range an important challenge for researchers (1). HDP is one of the important causes of maternal and neonatal-perinatal death and other serious adverse pregnancy outcomes worldwide and has been a focus of attention for clinicians for many years because of its wide range of effects as well as high medical expenditure. A variety of studies have investigated the relationship between maternal subclinical hypothyroidism and a variety of obstetric as well as neonatal outcomes including HDP (4–7). Studies have shown impaired endothelium-associated vasodilation in patients with subclinical hypothyroidism (8), suggesting that subclinical hypothyroidism may be a risk factor for HDP. However, some existing clinical studies have conflicting conclusions and no uniform consensus has been reached.

In 2011, the American Thyroid Association (ATA) developed a unified standard for the diagnosis and treatment of thyroid disease during pregnancy (9). The guidelines recommend that every effort should be made to establish pregnancy-based reference ranges for serum TSH to accurately screen for SCH. When pregnancy and assay-specific TSH reference ranges are not available, the upper limit of 2.5 mIU/L in the first trimester and 3.0 mIU/L in the second trimester can be used. Based on the study of sample and ethnicity, the ATA guideline was revised in 2017 to include 4 mIU/L as the upper limit of normal for serum TSH values in early pregnancy (1).

Up to now, there are various studies on whether maternal subclinical hypothyroidism is associated with HDP. Some studies believe that women with subclinical hypothyroidism in pregnancy are at risk of HDP compared with euthyroid pregnant women during pregnancy (10, 11). However, the results of a META analysis showed no correlation between subclinical hypothyroidism in pregnancy and HDP (12). More importantly, since the ATA guidelines were revised in 2017, several studies have been published successively. Therefore, the purpose of this study was to systematically review the published eligible studies to determine the correlation between subclinical hypothyroidism during pregnancy and HDP, and to perform a more detailed analysis according to the difference in TSH cut-off values and different pregnancy periods, to provide a basis for clinical diagnosis and prognosis of the disease.

## Materials And Methods

### Search Strategy

Two reviewers were assigned to assess the eligibility of the literature search in the MEDLINE (PubMed), Cochrane Central, EMBASE, Web of Science, and SCOPUS databases between January 1949 and October 2021. Additionally, each reviewer re-assessed the relevance of the studies found for inclusion in the present study. We used the terms “pregnancy induced hypertension” [All Fields] OR “gestational hypertension” [All Fields]) OR “pregnancy transient hypertension” [All Fields]) OR “Preeclampsia” [All Fields]) OR “hypertensive disorder of pregnancy”. These previously mentioned terms were combined with AND (“subclinical hypothyroidism” [All Fields] OR “subclinical thyroid dysfunction” [All Fields]) OR “untreated subclinical hypothyroidism” [All Fields]) OR “maternal subclinical hypothyroidism” [All Fields] OR “thyrotropin “[Mesh Term]) OR “thyroid-stimulating hormone“ [All Fields]) OR “thyroid stimulating hormon” [All Fields]) OR “TSH” [All Fields]) OR ldquo; thyreotropin” [All Fields]) OR “thyrotropic hormone” [All Fields]) OR “maternal TSH level” [All Fields]). The references of all included original articles were also determined by two researchers for their eligibility. All controversial original articles were decided in consultation with a third study person.

### Study Selection and Eligibility Criteria

**Figure 1:**
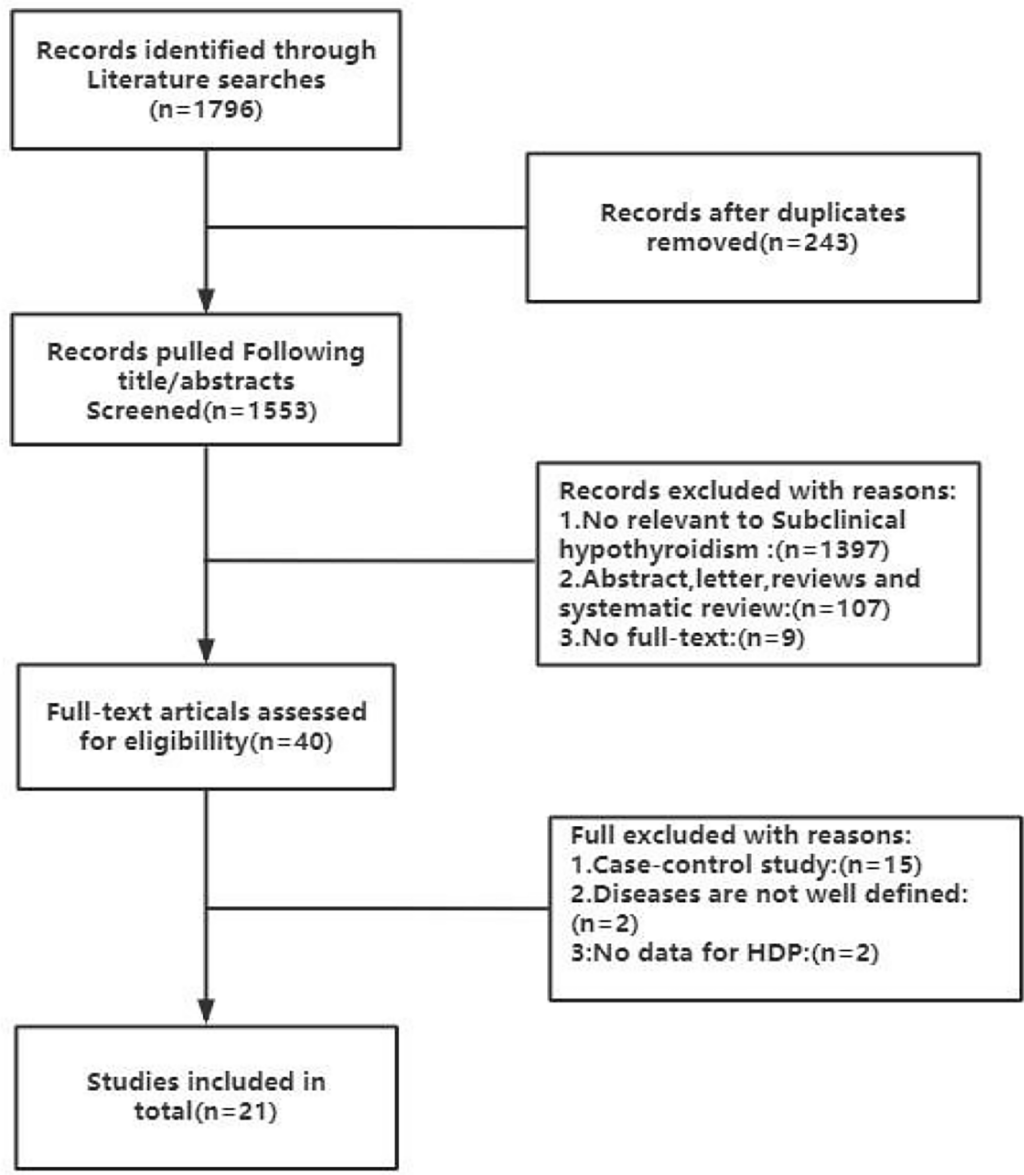
Flow chart of the literature search and selection process in the MEDLINE (PubMed), EMBASE, Cochrane Central, Web of Science and SCOPUS.

The inclusion criteria comprised of the following conditions: 1) articles that were published in English and were clinical cohort studies were eligible 2) studies needed to describe the specific gestational age and blood sample collection information 3) studies needed to provide the normal reference range of TSH and FT4, thyroglobulin status (if tested) and the kits used for detection 4) Studies needed to offer diagnostic criteria for gestational hypertension and preeclampsia. The exclusion criteria included: 1) randomized controlled study, cross-sectional studies, case-control studies, randomized controlled study case reports or reviews 2) full text not available. **Figure 1** is the flow chart of literature screening. Subclinical hypothyroidism during pregnancy was defined as serum TSH greater than the upper limit of the pregnancy-specific reference range and serum FT4 within the pregnancy-specific reference range. Gestational hypertension was defined as systolic blood pressure ≥ 140 mmhg and/or diastolic blood pressure ≥ 90 mmhg found after 20 weeks of gestation and required at least two blood pressure measurements in the same arm before diagnosis. Preeclampsia was defined as the presence of positive random urine protein or 24-hour urine protein ≥ 0.3g in addition to the above findings.

### Quality Assessment

The Newcastle-Ottawa Scale (NOS) was used to evaluate the quality of the literature in all finally included studies (13). The Newcastle-Ottawa Scale (NOS) mainly contains three parts, which are selectivity, comparability and outcome, with a maximum score of 9 stars. In the evaluation process, the objection shall be jointly decided by negotiation with a third party.

### Statistical Analysis

All statistical analyses were performed by STATA15.1. Random effects models were applied to calculate odds ratios (ORs) and 95% confidence intervals (CIs) to determine the association between subclinical hypothyroidism during pregnancy and HDP. The I²statistic was applied to test for heterogeneity between studies (14). When I² was less than 25%, it was considered that the heterogeneity between studies was low, and when I² value was greater than 75%, it indicated that the heterogeneity between studies was high. Subgroup analysis was used to investigate the source of heterogeneity between studies. First, without considering gestational age, TSH cut-off values of 3 mIU/L, and 4 mIU/L were used for analysis, respectively. Subsequently, different pregnancy periods (first trimester(T1):≤12weeks, second trimester (T2): ≤28weeks and third trimester(T3):>28weeks) were analyzed. When the original study tested serum TSH values separately in different pregnancy periods, if the TSH diagnostic threshold used was similar (i.e., TSH diagnostic cut-off values were > 3 mIU/L or < 3 mIU/L, and TSH diagnostic threshold values were > 4 mIU/L or < 4 mIU/L), the comparisons were performed according to the individual studies, otherwise the comparisons were performed separately. Sensitivity analyses were performed by examining the effects of individual studies as well as by employing different effect models. Publication bias was detected by the harbord test (15). This study follows PRISMA2009.

## Results

### Search Results

The literature screening process is summarised in **Figure 1**. Eventually, a total of 1796 articles were retrieved in the database according to the established search strategy, 243 articles were removed due to duplication. Subsequently, during screening through the abstract, 1397 studies unrelated to subclinical hypothyroidism were found, 107 reviews, letters and abstracts were excluded, and the other 9 original studies could not be obtained in full text and were eliminated. We then further searched the full texts of 40 articles to assess their eligibility, of which 15 were case-control studies, 2 studies were poorly defined for disease, and 2 studies that did not provide HDP-related data were excluded. Eventually, we included 22 original studies to investigate the relationship between subclinical hypothyroidism in pregnancy and HDP (7, 10, 11, 16–33).

### Characteristics of Qualified Literatures

**Table 1** depicts the basic features of the included articles. A total of 108831 patients from 10 countries from 2005 to 2020 were finally analyzed in this study. A total of 4808 patients with subclinical hypothyroidism, 94306 with euthyroidism, and the rest with other thyroid diseases [there is a part of the repeated population because Li, M.F., et al. and his colleagues assessed the same population with different diagnostic criteria, respectively (10)].There were 15 prospective cohort studies (16–20, 22–29, 31, 32) and 7 retrospective cohort studies (4, 7, 10, 11, 21, 30, 33). In addition, due to slight differences in thyroid parameters during different pregnancy periods, there were 10, 6, and 1 studies evaluating the relationship between subclinical hypothyroidism in pregnancy and HDP in the first, second, and third trimesters, respectively. However, only 2 studies have assessed the relationship between subclinical hypothyroidism and HDP in different TPOAb status (7, 25). Only five studies provided data on the development of HDP after treatment with levothyroxine in patients with subclinical hypothyroidism (11). Due to differences in sample size and study population, the incidence of subclinical hypothyroidism varied from 2.2% to 45.4%. In this study, the incidence of clinical hypothyroidism was 4.42%, the incidence in the first trimester was 8.17%, and the incidence in the second and third trimesters was 3.27% [calculated using the data obtained according to the 2017ATA guideline as the diagnostic criteria in the study by Li, M.F., et al. and his colleagues (10)]. The upper normal cut-off for TSH in this study was between 2.5 mIU/L and 5.78 mIU/L. Curiously, TSH values between 2.5 mIU/L and 4.08 mIU/L were defined as “mildly elevated TSH” in the study by Zhang et al, so they were analyzed separately in this study (7). The results of the quality evaluation of the included studies are presented in **Supplementary Table 1**. The results showed that all studies achieved high scores, suggesting high confidence in the meta-analysis results.

**Table 1:** The general characteristics of the 22 included studies.

Author	Year	Study type	Country	Sample size	The prevalence of SCH	Time points of assessment of thyroid parameters	The cut-off for TSH in SCH (mIU/L)
**Sitoris G. et al. (** **3** **)**	2020	prospective cohort study	Belgium	1521	10.45%	<G20w	>2.51
**Li M.F. et al. (** **10** **)**	2020	retrospective cohort study	China	1556	37.6% (2011ATA), 9.77% (2017ATA)	T1	>2.5 (2011ATA), >4 (2017ATA)
**Lai H., Z.Y. et al. (** **26** **)**	2020	prospective cohort study	China	1226	5.79%	T1	>3.0
**Wu M.Q. et al. (** **11** **)**	2019	retrospective cohort study	China	6157	2.68%	T1, T2	>4.432 (T1),>4.053(T2)
**Cakmak et al. (** **4** **)**	2019	retrospective cohort study	Turkey	8916	10.43%	T1	>2.5
**Gupta R. et al. (** **23** **)**	2018	prospective cohort study	India	1268	11.20%	<G20w	T1:>2.5 T2:>3.0
**Furukawa S. et al. (** **21** **)**	2017	retrospective cohort study	Japan	745	22.41%	<G20w	>3
**Hebbar S. et al. (** **24** **)**	2017	prospective cohort study	India	171	45.40%	T1	>2.5
**Zhang et al. (** **7** **)**	2016	retrospective cohort study	China	3562	1.67%	T1	>4.08
**Kishore R. N.et al. (** **32** **)**	2015	prospective cohort study	India	263	6.08%	T1	>2.5
**Ajmani S.N. et al. (** **16** **)**	2014	prospective cohort study	India	400	9.00%	T2	>3.0
**Chen L.M. et al. (** **19** **)**	2014	prospective cohort study	China	8012	4.63%	T1,T2,T3	>3.47 (T1),>3.81(T2),>4.99(T3)
**Saki F. et al. (** **29** **)**	2014	prospective cohort study	Iran	600	11.30%	T2	>3
**Breathnach F.M. et al. (** **17** **)**	2013	prospective cohort study	Ireland	904	1.77%	<G20w	>4.1
**Goel P. et al. (** **22** **)**	2012	prospective cohort study	India	1005	3.40%	T1,T2,T3	T1>5.0,T2>5.78,T3:5.7
**Wang S. et al. (** **30** **)**	2012	retrospective cohort study	China	756	26.49%	T1	>2.5
**Karakosta P. et al. (** **25** **)**	2012	prospective cohort study	Greece	1170	6.92%	<G20w	T1:>2.53 T2:>2.73
**Wilson K.L. et al. (** **33** **)**	2012	retrospective cohort study	USA	24883	2.12%	<G20w	>4.13
**Mannisto et al. (** **27** **)**	2010	prospective cohort study	Finland	5805	3.90%	<G20w	>3.6
**Sahu M.T. et al. (** **28** **)**	2010	prospective cohort study	India	633	6.47%	T2	>5.5
**Cleary-Goldman J. et al. (** **20** **)**	2008	prospective cohort study	USA	21980	2.20%	T1,T2	>4.29 (T1),>3.94(T2)
**Casey B.M. et al. (** **18** **)**	2005	prospective cohort study	USA	17298	2.33%	<G20w	>2.74

### Meta-Analysis

**Figure 2:**
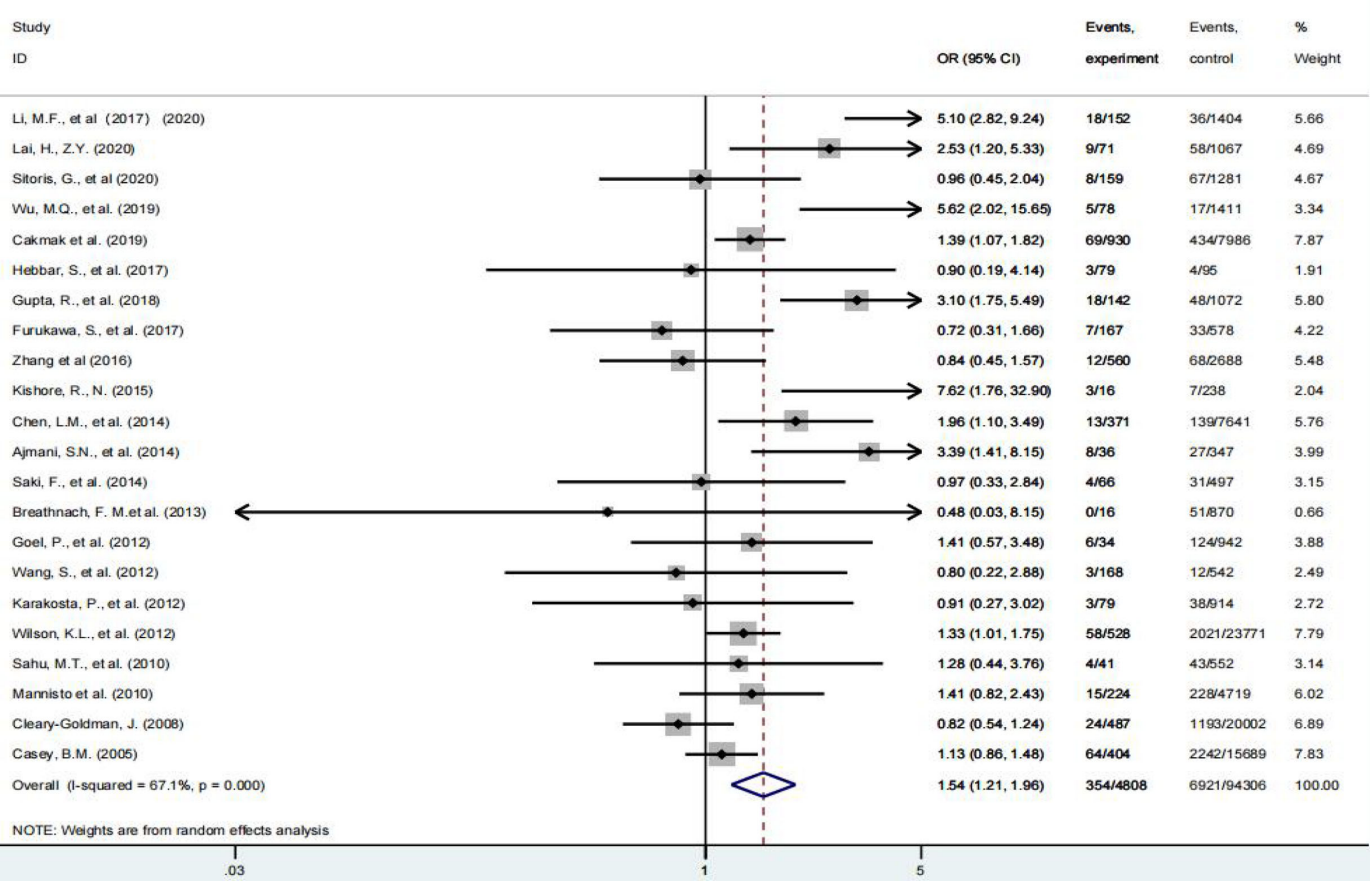
Forest plot of relative risk and 95% confidence interval (CI) of pooled studies comparing pregnant women with subclinical hypothyroidism to euthyroid pregnant women for risk of HDP.

Fifty percent of the 22 studies included in this paper believed that subclinical hypothyroidism in pregnancy was associated with HDP, and the rest were considered unrelated. As shown in **Figure 2**, compared with euthyroidism, pregnant women with subclinical hypothyroidism had an increased risk of HDP [OR = 1.54(95% CI: 1.21-1.96) I²=67.1%]. Disappointingly, five studies further investigated these patients treated with levothyroxine did not have a reduced risk of HDP compared with patients with subclinical hypothyroidism who were not treated with levothyroxine (p = 0.241) (7, 11, 30, 34, 35), however, due to the limited number of current studies, the credibility of the conclusions is limited.

### Subgroup Analysis

**Figure 3:**
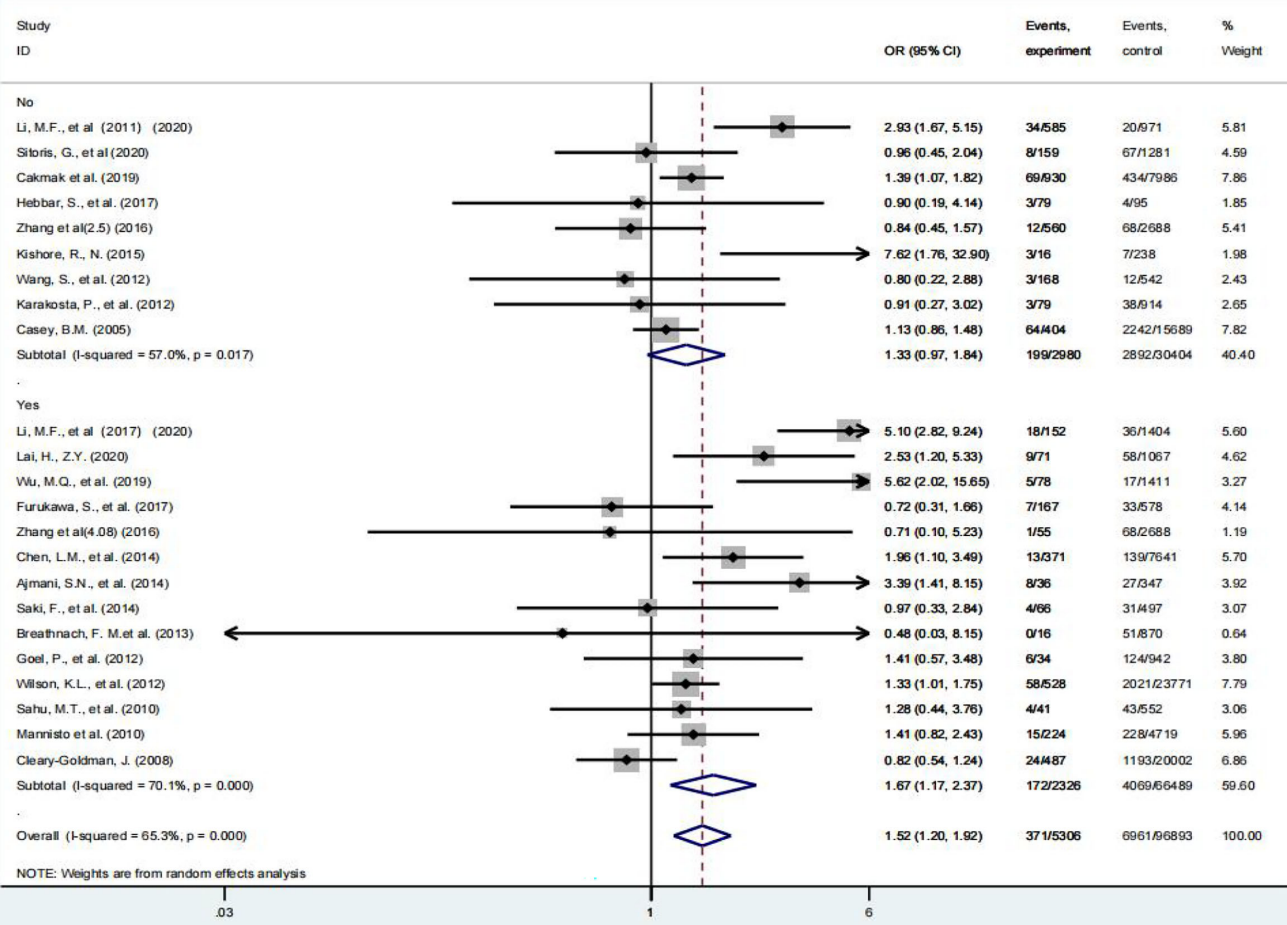
Forest plot of relative risk and 95% CI of pooled studies comparing pregnant women with subclinical hypothyroidism to euthyroid pregnant women for risk of HDP that used a TSH upper limit of 3.0 mIU/L. and (B) that used a TSH upper limit of 4.0 mIU/L.

**Figure 4:**
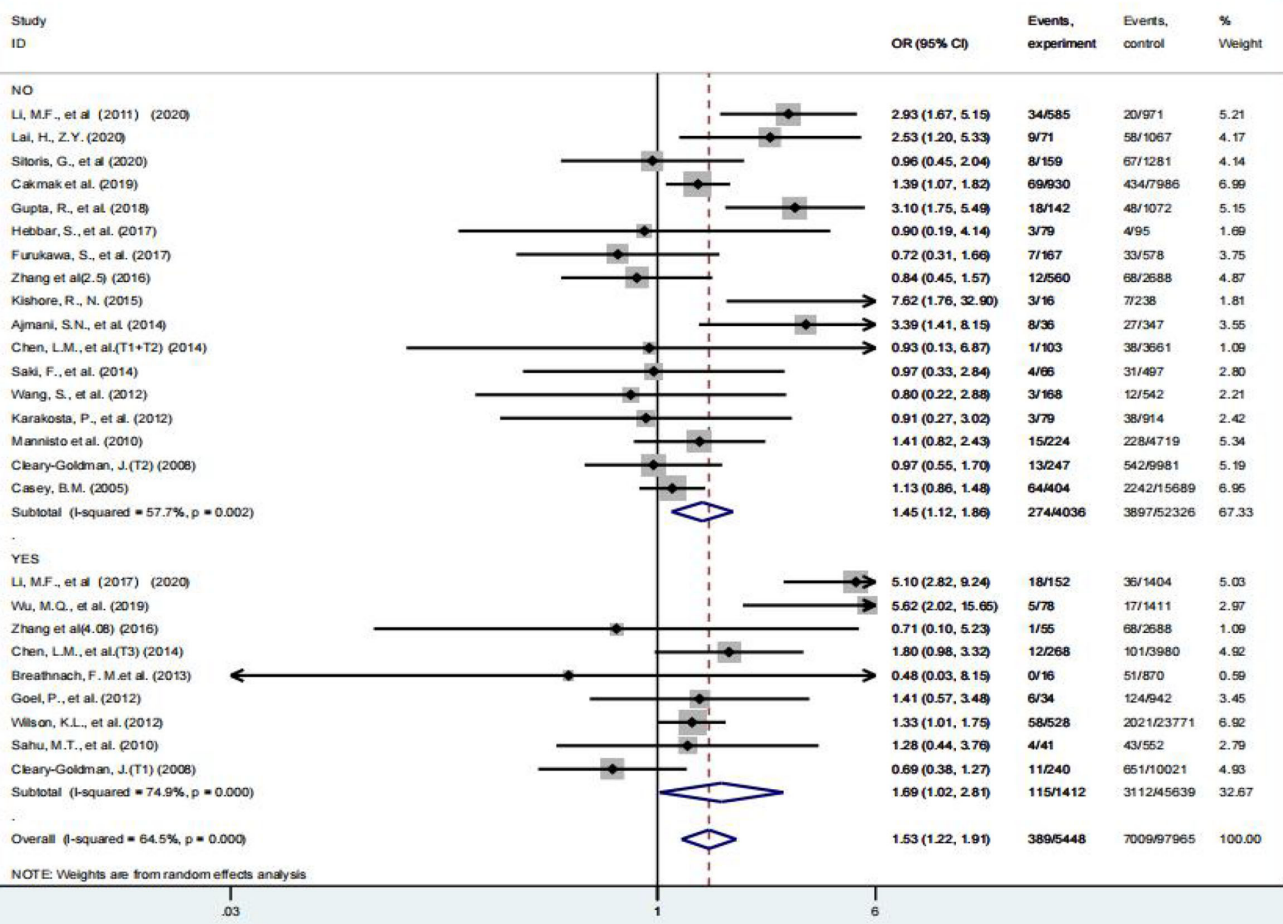
Forest plot of relative risk and 95% CI of pooled studies comparing pregnant women with subclinical hypothyroidism to euthyroid pregnant women for risk of HDP that used a TSH upper limit of 4.0 mIU/L.

According to the 2017ATA guideline, 4.0 mIU/L can be used as the upper limit of TSH in the first trimester when the specific TSH reference range is not available, and 3.0 mIU/L can be used as the upper limit of TSH in the second and third trimesters according to the 2011ATA guideline. Therefore, in this study, 3.0 mIU/L and 4.0 mIU/L were used as TSH diagnostic cut-off values to complete the grouping analysis, respectively, regardless of the effect of gestational age. As shown in **Figures 3**, **4**, when a meta-analysis was performed at a TSH diagnostic cut-off above or below 3.0 mIU/L(Because the study subjects were not clearly distinguished by a TSH diagnostic cut-off of 3 mIU/L in the study by Gupta, R (23), this study was excluded from the subgroup analysis.), SCH was not associated with HDP at TSH diagnostic cut-off of less than 3.0 (P = 0.077), and the risk of developing HDP was increased 1.67-fold (95% CI: 1.17 – 2.37) at TSH diagnostic cut-off of more than 3.0 mIU/L. Curiously, when a meta-analysis was performed using a TSH diagnostic cut-off of 4.0 mIU/L as a grouping basis, patients with subclinical hypothyroidism in pregnancy had a 1.69-fold (95% CI: 1.02 – 2.81) increased risk of HDP above this threshold compared with euthyroid pregnant women, and a 1.45-fold (95% CI: 1.12 – 1.86) increased risk below this threshold (**Table 2**).

**Table 2:** Results of subgroup analysis.

Parameter	Category	No.of study	OR (95%CI)	I²	P
TSH≥3mIU/L	Yes	14	1.67 (1.17-2.37)	70.10%	0.004
	No	9	1.33 (0.97-1.84)	57.00%	0.077
TSH≥4mIU/L	Yes	9	1.69 (1.02-2.81)	74.90%	0.004
	No	17	1.45 (1.12-1.86)	57.70%	0.043
Pregnancy period	first trimester	10	1.79 (1.04-3.07)	77.60%	0.034
	second and third trimester	6	1.70 (1.05-2.75)	48.40%	0.030

**Figure 5:**
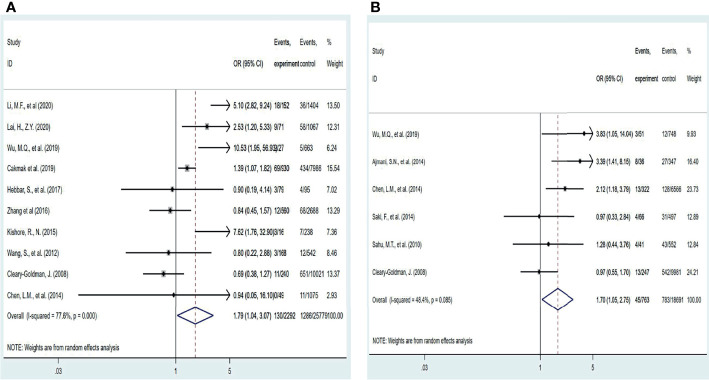
Forest plot of relative risk and 95% CI of pooled studies comparing pregnant women with subclinical hypothyroidism to euthyroid pregnant women for risk of HDP **(A)** gestational age at a screening at the first trimester and **(B)** gestational age at a screening at second and third trimester.

However, the effect of different pregnancy periods on thyroid parameters cannot be ignored either. Therefore, we further investigated the relationship between screening diagnosis of subclinical hypothyroidism in the first or second and third trimester of pregnancy and the development of HDP(**Figure 5**). The results suggest that the risk of HDP is increased by 1.79-fold (95% CI: 1.04 – 3.07) after screening in the first trimester for the diagnosis of subclinical hypothyroidism and by 1.58-fold (95% CI: 1.03 – 2.42) during the second and third trimesters of pregnancy (**Table 2**). Only 2 studies provided data on the effect of TPOAb status on the development of HDP and were not analyzed in this meta-analysis.

### Sensitivity Analysis and Publication Bias

First, in this study, model stability was judged by different effect models. Second, the stability of the conclusions was judged by investigating the effects of individual studies one by one according to different effect scales, and the above results suggested that the conclusions of this study were stable and credible (results shown in **Supplementary Figure 1** and **Figure 2**). We used the harbord test to detect publication bias and found no significant publication bias (P = 0.081).

## Discussion

Up to now, a total of 22 articles have explored the relationship between subclinical hypothyroidism during pregnancy and HDP, but the findings are not consistent. To our knowledge, the results of a meta-analysis by Maraka, S., et al. suggested that subclinical hypothyroidism during pregnancy was not associated with gestational hypertension and preeclampsia (12). However, some relevant studies have been published recently, proposing some new conclusions. The results of two recently completed prospective cohort studies indicate that subclinical hypothyroidism in pregnancy is associated with an increased risk of developing HDP and is a risk factor for HDP (26–31). Similarly, the study by Cakmak and Wu, M.Q reached the same conclusion (4, 11). Therefore, we reviewed a total of 108831 patients involved in 10 countries in the relevant published literature to re-evaluate the correlation between subclinical hypothyroidism during pregnancy and HDP, so as to guide clinical practice and improve adverse pregnancy outcomes in perinatal pregnant women.

Due to differences in diagnostic cut-off selection, race, iodine intake, etc., the prevalence of subclinical hypothyroidism during pregnancy ranges from 1.5% to 42.9% (3), and the incidence is about 10 times that of overt hypothyroidism (36). Therefore, the huge sick population has also gained the attention of the majority of researchers and become a hot spot and frontier of current clinical and basic research. Compared with overt hypothyroidism, the effect of subclinical hypothyroidism during pregnancy on adverse pregnancy outcomes is not clear. Existing research suggests that patients with subclinical hypothyroidism in pregnancy may have an increased risk of gestational diabetes, spontaneous abortion, and preterm delivery compared with euthyroid pregnant women (37–39). In addition, short-term neurodevelopment as well as long-term mental development, and motor development may also be affected in offspring (40, 41). Previous studies have suggested that thyroid hormone has a profound effect on the cardiovascular system through cardiac contraction, systemic vascular resistance, and cholesterol metabolism (42–44), which induces the production of NO under the action of ion channels, which in turn produces impaired endothelium-dependent vasodilation and the formation of hypertensive disorders (8). However, HDP is a common complication that seriously threatens maternal and child health and safety is one of the important causes of maternal death. It has become a disease that obstetricians focus on screening and treatment. Therefore, it is of great practical significance to clarify the relationship between subclinical hypothyroidism in pregnancy and HDP to standardize pregnancy management and reduce adverse pregnancy outcomes in pregnant women.

Of the 22 articles included in this study, about 50% of the studies considered subclinical hypothyroidism in pregnancy to be unrelated to HDP. Ultimately, our findings showed a 1.54-fold increased risk of HDP in pregnant women with subclinical hypothyroidism compared to euthyroid pregnant women. Considering the heterogeneity among studies caused by different diagnostic cut-off values selected in different studies, we further used TSH diagnostic cut-off values of 3.0 mIU/L and 4.0 mIU/L as a grouping basis for analysis. The results showed that when the diagnostic cut-off value of TSH was less than 3 mIU/L, subclinical hypothyroidism in pregnancy was not associated with HDP, and when it was more than 3 mIU/L, the risk of HDP increased by 1.67 times. When the TSH diagnostic cut-off value is 4 mIU/L, above or below this threshold, the risk of developing HDP increases. Although only 9 studies were above the threshold, the risk of developing HDP was still 1.69 times, which was highest in all subgroup analyses. This is consistent with the newly recommended diagnostic cut-off value of 4 mIU/L for TSH by the ATA. This conclusion may be more supported as more clinical studies are conducted in the future.

Pregnancy status profoundly affects thyroid function as well as the metabolism of thyroid hormones, making them significantly different from non-pregnant periods. Because human chorionic gonadotropin and TSH have similar chemical structures, they have partial TSH function, which in turn directly affects the thyroid function of pregnant women and increases the complexity and heterogeneity of thyroid diseases during pregnancy. Generally, the serum HCG level increases and TSH level decreases from 8 to 14 weeks of gestation, TSH decreases to the lowest level from 10 to 12 weeks of gestation, gradually increases in the second trimester, and is even higher than that of the general population in the third trimester (1). Thus, thyroid function parameters are constantly changing in different pregnancy periods, rather than being static. Therefore, it is very necessary to observe the relationship between subclinical hypothyroidism in pregnancy and HDP based on different pregnancy periods. Our study concluded that whether subclinical hypothyroidism was diagnosed in the first or second and third trimester of pregnancy, the risk of HDP increased by more than 1.5 times in the future, but this risk did not decrease after levothyroxine treatment (OR: 1.27 95% CI: 0.85 – 1.88), probably since only five studies have provided data on the development of HDP after the application of levothyroxine for subclinical hypothyroidism. Similarly, Yamamoto’s study found that adverse pregnancy outcomes (miscarriage, gestational hypertension, preeclampsia, etc.) were not improved in pregnant women treated with thyroxine (45), so there is currently insufficient evidence to prove that patients can benefit after treatment with thyroxine. Similarly, ATA guidelines also recommend serum TSH testing only for pregnant women with risk factors for hypothyroidism, rather than universal screening (1). Of course, the conclusions of these studies are only based on a small number of original studies, and their real clinical value still needs to be proved by more subsequent clinical studies.

The main limitation of this study is that most of the studies did not describe thyroid autoantibody status, however, thyroid autoantibodies may be associated with a variety of adverse pregnancy outcomes (46), thus leading to an underestimation of the true impact of subclinical hypothyroidism on HDP. It is well-known that iodine intake is the influencing factor of subclinical hypothyroidism, but the selection of evaluation indicators of iodine nutrition status during pregnancy is still a difficult point in current clinical practice, so most studies do not provide specific iodine nutrition status of pregnant women, which may be another limitation of this study. In addition, in the studies conducted after the publication of the new guidelines in 2017, only one study included the diagnostic cut-off value of TSH recommended in the guidelines as the diagnostic criteria so that the results of this analysis were not very significant. In addition, because some studies did not describe whether the included study subjects excluded the intervention of thyroid drugs so that we could not estimate the effect caused by these confounding factors, combined with the existing study results, we do not recommend the treatment of thyroxine for pregnancy with subclinical hypothyroidism. Although geographic and ethnic diversity in TSH concentrations during pregnancy does exist, guidelines state that the availability of calculated reference ranges for specific pregnancies is limited for most ethnic and populations with adequate iodine intake and no thyroid autoantibodies (1). Therefore, in order to provide guidance to all patients and clinicians, the use of specific reference ranges and cut-off values also has important practical implications, although there may be some bias.

## Conclusion

The results of this meta-analysis indicate that subclinical hypothyroidism in pregnancy is associated with an increased risk of developing HDP, and this association exists regardless of the gestational period. However, the available evidence cannot support these patients receiving thyroxine intervention can benefit from it, so routine screening is only recommended for pregnant women with risk factors for hypothyroidism. In the future, we hope to carry out more scientific and rigorous clinical studies to clarify the relationship between subclinical hypothyroidism and HDP to improve patient prognosis.

## Data Availability Statement

The original contributions presented in the study are included in the article/**Supplementary Material**. Further inquiries can be directed to the corresponding authors.

## Author Contributions

YH: Protocol development, Data collection or management, Data analysis, Manuscript writing and editing. JW: Manuscript writing and editing. XW: Data collection or management. LO: Data analysis, Manuscript editing. YL: Protocol development, Data analysis, Manuscript editing.

## Conflict of Interest

The authors declare that the research was conducted in the absence of any commercial or financial relationships that could be construed as a potential conflict of interest.

## Publisher’s Note

All claims expressed in this article are solely those of the authors and do not necessarily represent those of their affiliated organizations, or those of the publisher, the editors and the reviewers. Any product that may be evaluated in this article, or claim that may be made by its manufacturer, is not guaranteed or endorsed by the publisher.
