# Supplementary material for: Relationship Between Subclinical Hypothyroidism in Pregnancy and Hypertensive Disorder of Pregnancy: A Systematic Review and Meta-Analysis
Source: Front Endocrinol (Lausanne). 2022 Mar 8;13:823710. doi: 10.3389/fendo.2022.823710 (PMC8959212; doi:10.3389/fendo.2022.823710)
Supplement: Supplementary file 1 [file DataSheet_1.docx]

**Supplementary Table 1：**The results of the quality evaluation of the included studies（1）


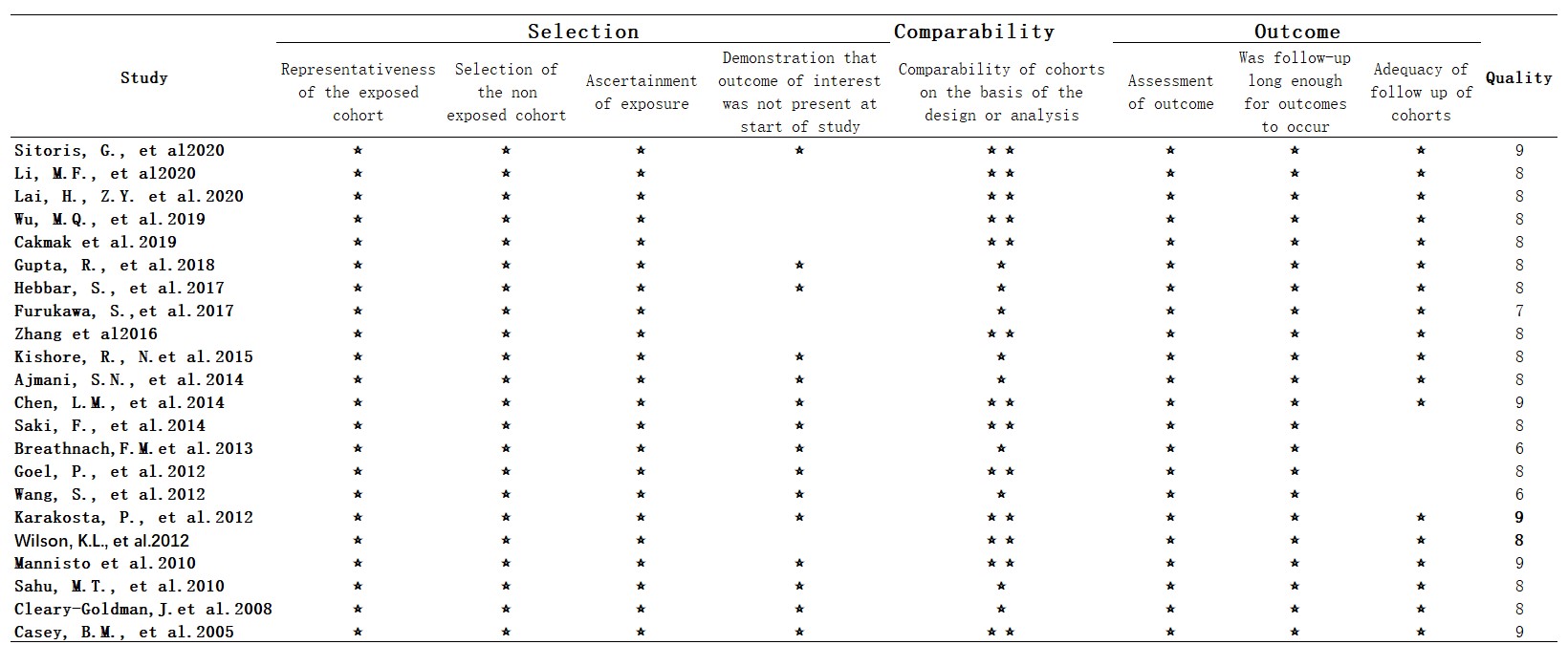


**Supplementary Fig 1：**model stability was judged by different effect models(2)


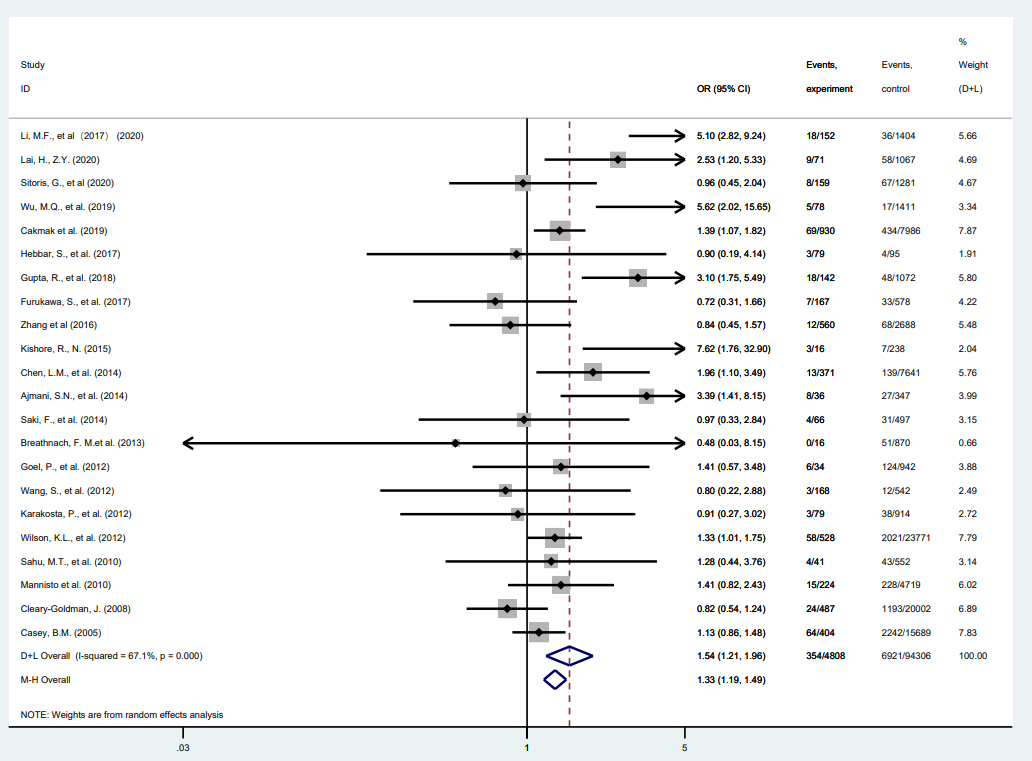


**Supplementary Fig2：**The results of sensitivity analysis was assessed by investigating the effects of individual studies one by one according to different effect scales.


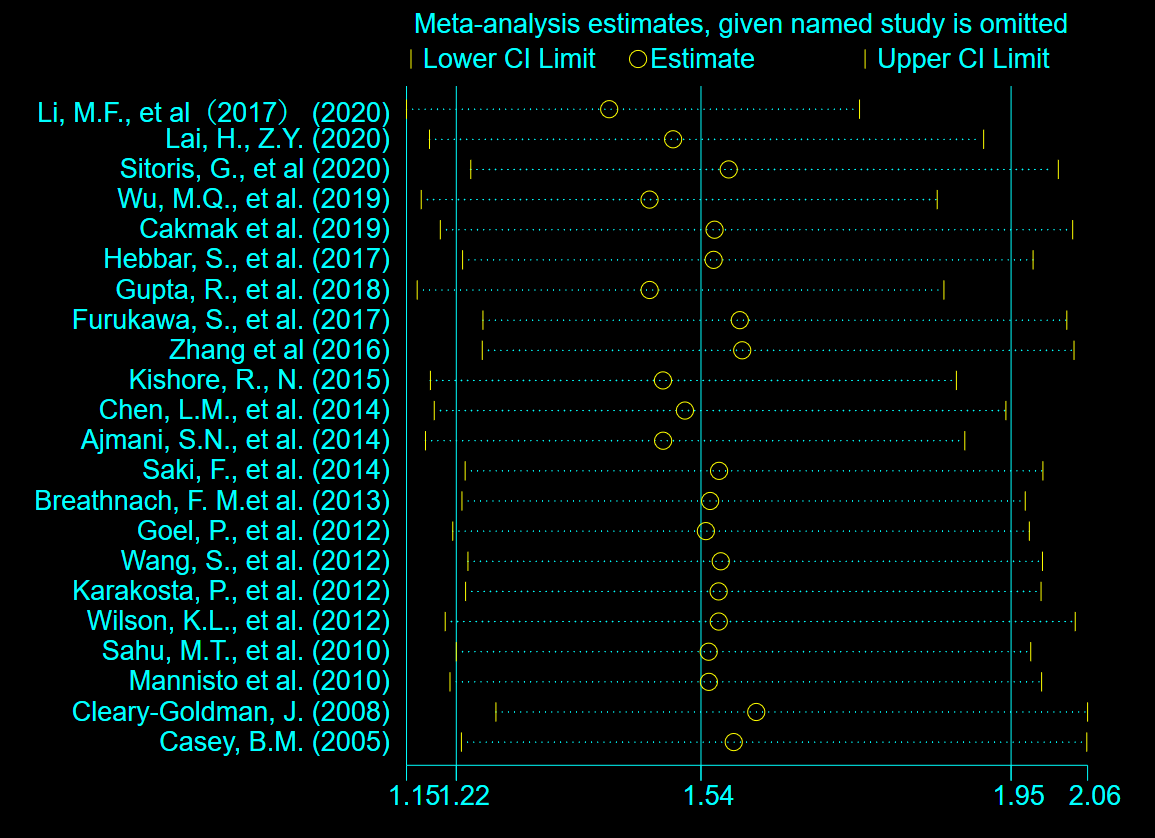


References:

1. Wells GA, Shea B, O’Connell D, Peterson J, Welch V, Losos M, et al., The Newcastle-Ottawa Scale (NOS) for assessing the quality of nonrandomized studies in meta-analyses. Available at: www.ohri.ca/programs/clinical_epidemiology/oxford.asp (accessed June 30, 2015)
2. DerSimonian R, Laird N 1986 Meta-analysis in clinicaltrials. Control Clin Trials 7:177–188.
